# Supplementary figures and images for: Potentially Health-Promoting Spaghetti-Type Pastas Based on Doubly Modified Corn Starch: Starch Oxidation via Wet Chemistry Followed by Organocatalytic Butyrylation Using Reactive Extrusion
Source: Polymers (Basel). 2023 Mar 29;15(7):1704. doi: 10.3390/polym15071704 (PMC10097208; doi:10.3390/polym15071704)

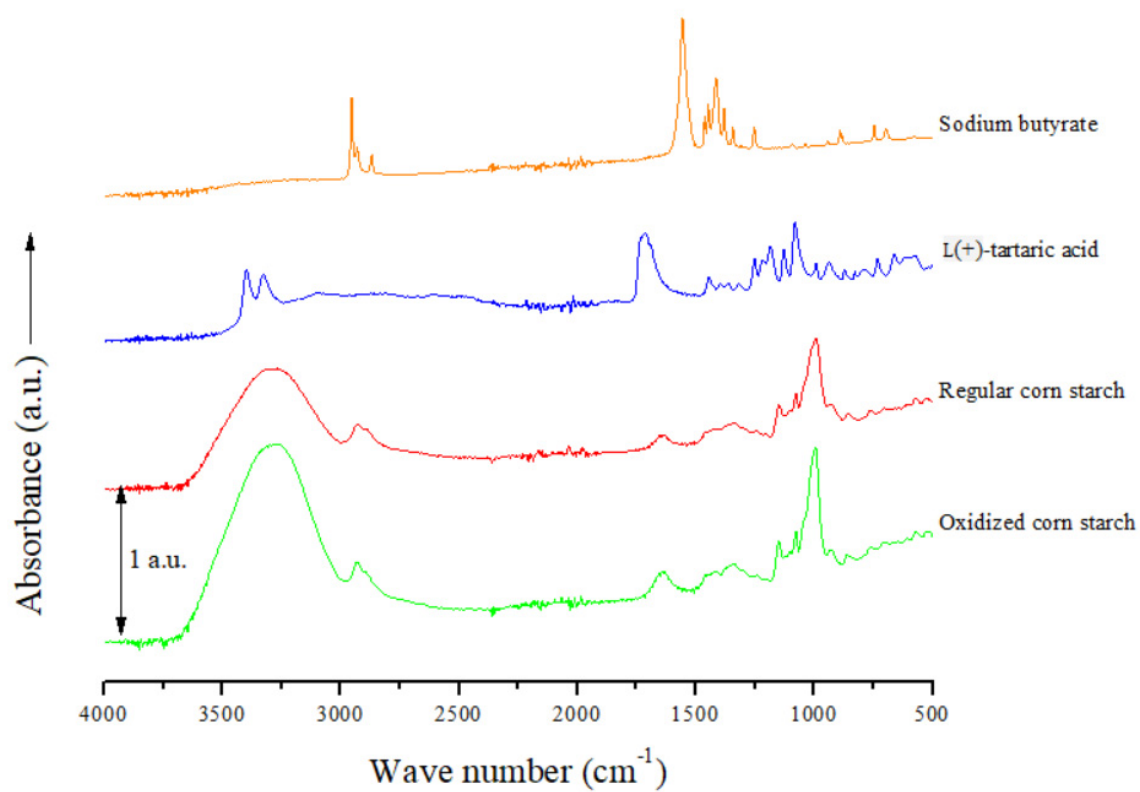

**Figure S1.** ATR/FTIR spectra of the different feedstocks used.

Supplement: Supplementary file 1 [file polymers-15-01704-s001.zip › Figure S1 (November 21, 2022).pdf]
